# High‐resolution quantitative MRI of multiple sclerosis spinal cord lesions

**DOI:** 10.1002/mrm.29152

**Published:** 2022-01-11

**Authors:** Amy R. McDowell, Natalia Petrova, Daniele Carassiti, Marc E. Miquel, David L. Thomas, Gareth J. Barker, Klaus Schmierer, Tobias C. Wood

**Affiliations:** ^1^ Queen Square Centre for Neuromuscular Diseases UCL London United Kingdom; ^2^ The Blizard Institute (Neuroscience, Surgery & Trauma), Queen Mary University of London, Barts and The London School of Medicine & Dentistry London United Kingdom; ^3^ Clinical Physics Barts Health NHS Trust London United Kingdom; ^4^ Department of Brain Repair and Rehabilitation UCL Queen Square Institute of Neurology London United Kingdom; ^5^ King’s College London London United Kingdom; ^6^ Clinical Board Medicine (Neuroscience) Barts Health NHS Trust The Royal London Hospital London United Kingdom

**Keywords:** multiple sclerosis, myelin water fraction, spinal cord

## Abstract

**Purpose:**

Validation of quantitative MR measures for myelin imaging in the postmortem multiple sclerosis spinal cord.

**Methods:**

Four fixed spinal cord samples were imaged first with a 3T clinical MR scanner to identify areas of interest for scanning, and then with a 7T small bore scanner using a multicomponent‐driven equilibrium single‐pulse observation of T_1_ and T_2_ protocol to produce apparent proton density, T_1_, T_2_, myelin water, intracellular water, and free‐water fraction maps. After imaging, the cords were sectioned and stained with histological markers (hematoxylin and eosin, myelin basic protein, and neurofilament protein), which were quantitatively compared with the MR maps.

**Results:**

Excellent correspondence was found between high‐resolution MR parameter maps and histology, particularly for apparent proton density MRI and myelin basic protein staining.

**Conclusion:**

High‐resolution quantitative MRI of the spinal cord provides biologically meaningful measures, and could be beneficial to diagnose and track multiple sclerosis lesions in the spinal cord.

## 1 INTRODUCTION

The myelin sheath is an extended and modified plasma membrane wrapped around axons in a spiral fashion.^1^ Myelin is composed of about 40% water, whereas the dry mass consists of about 80% lipids, primarily the glycolipid galactocerebroside, and 20% protein.^2^ Due to its importance for normal function of the nervous system, and severe consequences of myelin damage in demyelinating diseases (such as multiple sclerosis [MS]), the noninvasive in vivo assessment of myelin remains a key focus of imaging research, not least in the era of regenerative medicine.^3^

The spinal cord is most commonly affected in people with MS at presentation.^4^, ^5^ Lesions in the spinal cord have been shown to predict both a diagnosis of MS in patients with radiologically isolated, as well as clinically isolated, syndromes of demyelination^6^, ^7^; they are also associated with more severe disability.^6^, ^8^ As MS evolves over time, much of the permanent and deteriorating disability in people with MS affects lower body functions, including lack of sphincter control, sexual dysfunction, and impaired leg movement and coordination, resembling the clinical syndrome of progressive myelopathy.^9^, ^10^, ^11^, ^12^ Metrics such as spinal cord cross‐sectional area have been shown to be associated with MS‐related disability in vivo.^13^ However, the cross‐sectional area is affected by numerous elements of MS pathology including inflammation, edema, myelin content, the number of axons and gliosis.

Magnetic resonance imaging is used widely in the assessment of MS, but standard qualitative T_1_‐weighted and T_2_‐weighted images are largely nonspecific for the underlying pathology.^14^ Quantitative MRI has the potential to distinguish specific tissue features, such as separating demyelination from axonal loss.^15^ Multicomponent relaxometry is a family of methods that can quantify the fractional amount of water in different tissue environments by exploiting the differences in relaxation time values. In the nervous system, these environments include free water in CSF, intracellular and extracellular water (IEW), and, most importantly in the context of demyelinating diseases, water “trapped” between the myelin bilayers. The ratio of myelin water signal to total water signal is termed myelin water fraction (MWF).^16^

Along with other related quantitative measures, including the relaxation times themselves, MWF has shown promise to identify specific features of MS pathology and monitoring disease progression.^17^, ^18^ The gold‐standard method for evaluating the MWF is analysis of a multi‐echo T_2_ relaxation curve.^16^ The multi‐echo T_2_ method has been previously compared to histology in *post mortem* brain at 1.5 T,^19^ and Laule et al demonstrated a strong correlation of MWF in MS tissue samples at 7 T with luxol fast blue staining intensity for myelin on histology.^20^ These studies support the hypothesis that myelin can be mapped in the brain using MWF. However, clinical translation of multi‐echo T_2_ has been hampered by the long acquisition time required.^21^

In contrast, gradient echo–based methods, such as the driven equilibrium single‐pulse observation of T_1_ and T_2_, are inherently fast.^22^ A multi‐component version of driven equilibrium steady‐state observation of T_1_ and T_2_ (mcDESPOT) has been used in clinical studies of MS,^23^ primary lateral sclerosis,^24^ and monitoring myelin development in children.^25^, ^26^, ^27^, ^28^, ^29^ However, histological validation has been lacking, and recent work has shown that mcDESPOT suffers from unpredictable bias, but as there was sufficient data for analysis, we present it here.^30^, ^31^

Given that significant efforts in MS research are directed toward development of strategies for myelin repair (remyelination),^32^ imaging biomarkers of myelin are desirable. To establish whether mcDESPOT could provide such an index (or indices), we undertook correlative experiments using postmortem spinal cord, to quantitatively compare MRI and histology data.

## 2 METHODS

This study was covered by the UK Multiple Sclerosis Tissue Bank approval (Research Ethics Committee reference number 08/MRE09/31). Formalin‐fixed spinal cords were placed on a Perspex frame and inserted into glass tubes. The formalin was drained and the tubes then filled with perfluoropolyether (Fomblin; Solvay Solutions, United Kingdom) to provide a susceptibility‐matched signal‐free background (Supporting Information Figure S1).

Qualitative structural MRI was performed on the whole cords at 3 T (Achieva Tx, Philips Healthcare, Best, Netherlands) using a 15‐element SENSE spine coil and a turbo spin‐echo sequence with the following parameters: TE = 16 ms, TR = 5000 ms, voxel size = 0.4 × 0.4 × 2.0 mm^3^). Nine areas of interest were identified by a neurologist with > 20 years of experience in reading MRI of people with MS (KS) for scanning at 7 T. The thoracic level 1 was marked with a radiologically opaque marker tied to the nerve root; measurements of distance from the marker for each lesion were noted and used to inform positioning of the glass tube for subsequent 7T scanning.

Next, the cords were scanned using a 7T preclinical scanner (Agilent Technologies, Santa Clara, CA) and 39‐mm diameter quadrature transmit‐receive RF coil (Rapid Biomedical, Rimpar, Germany) designed for whole‐body rodent imaging. As the human spine is longer than the achievable FOV with this setup, for each area of interest the glass tubes were moved in or out of the RF coil as required to place the desired area at both the center of the RF coil and the isocenter of the magnet. We observed that the active area of the RF coil was longer than the linear region of the image encoding gradients. As the samples were long enough to protrude into this region, protons that were excited by the RF but not encoded by the gradients resulted in a “zipper artifact” in some images. This was minimized by careful rotation of the samples so that the artifacts did not intrude into areas required for analysis. In most samples, a small quantity of formalin remained after draining, which produced bright signal at the top of the sample tube. To avoid this wrapping into the tissue, the FOV was increased in the vertical direction.

Spoiled gradient‐echo and balanced SSFP (with two RF phase cycles) sequences were acquired with the parameters given in Table 1, along with an actual flip‐angle acquisition for B_1_ correction.^33^ Images were resampled to 150 × 150 × 300 μm^3^ for processing. The images were processed using the open‐source Quantitative Imaging Tools (QUIT) C++ toolbox^34^ (available from http://github.com/spinicist/QUIT). T_1_ maps were generated using nonlinear least‐squares fitting to images with all flip angles,^35^, ^36^ and T2 and apparent proton density (aPD) maps were generated using the driven equilibrium single pulse observation of T(2) with full modelling method.^37^

**TABLE 1:** Magnetic resonance imaging sequence acquisition parameters

Sequence	TE/TR (ms)	Flip angles	Voxel size (μm^3^)	Matrix size
SPGR	11.3/25	6, 8, 10, 15, 20, 25, 35, 45	100 × 125 × 200	160 × 160 × 160
bSSFP	2/4	14, 17, 20, 25, 35, 50, 65	100 × 125 × 200	160 × 160 × 160
AFI	2.62/7.5	55	250 × 250 × 500	64 × 64 × 64

Abbreviations: AFI, actual flip angle; bSSFP, balanced SSFP; SPGR, spoiled gradient echo.

Calculation of MWF from mcDESPOT requires the a priori specification of valid initial ranges of T_1_ and T_2_ for each water component.^30^, ^38^ It has been recognized that in postmortem tissue these relaxation times change following fixation.^39^, ^40^, ^41^ The single‐component T_1_ and T_2_ of white matter (WM) in a healthy cord was used as a reference point for the myelin water T_1_ and T_2_; healthy gray‐matter (GM) relaxation times were used as reference points for the intracellular/extracellular water T_1_ and T_2_; and literature values were used for the free‐water fraction.^38^ Fitting parameters including the ranges used for each water component are given in Table 2. Non‐overlapping ranges that encompassed the reference points or literature values for these parameters were used. It has been shown that neglecting the effect of TE in the spoiled gradient‐echo and balanced SSFP signal models lead to large errors in derived MWF values, so the TE‐corrected signal model was used.^42^

**TABLE 2:** Minimum and maximum values that were set for T_1_ and T_2_ of each component during mcDESPOT fitting

	MWF	IEWF	FWF
T_1_ (ms)	50–350	400–800	1000–5000
T_2_ (ms)	8–14	14–25	200–1500
Fraction constraints (%)	0.1–50	NA	0.1–99
Myelin residence time (ms)	10–250	NA	NA

Note

The expected T_1_ and T_2_ of MWF and IEWF were set significantly lower than for an equivalent in vivo experiment because formalin fixation is known to shorten these values. Fraction constraints constrain the MWF and FWF to within expected values to prevent convergence to an erroneous local minima. Myelin residence time is the mean time that a water molecule stays in the myelin pool before exchanging to the intracellular/extracellular water pool.

Abbreviation: mcDESPOT, multi‐component version of driven equilibrium steady‐state observation of T_1_ and T_2_.

The MR parameter maps were then matched with histology using positional information (cord level and distance of the lesion from the marker) from the 3T whole‐cord scans, and checked against anatomical features (such as lesion shape and gray‐matter anatomy) in the histology and 7T images. MIPAV (http://mipav.cit.nih.gov/) was used to manually define regions of interest (ROIs) on aPD maps and transfer them to all other maps for ROI analysis.

For histology, cords were dissected axially into blocks of 5‐mm thickness. Each tissue block was then marked with tissue dye to retain information on its spatial orientation and processed for embedding in paraffin. Ten‐micromillimeter‐thick sections were cut from each tissue block using a Shandon Finesse ME1 microtome (Thermo Scientific, United Kingdom). Care was taken to cut the sections in a plane perpendicular to the anterior spinal artery. The sections were mounted on Superfrost1 slides (VWR, United Kingdom) and left in a 608°C oven overnight.

Serial sections of each block were stained for hematoxylin and eosin, phosphorylated neurofilaments (SMI‐31, mouse monoclonal, 1:1000; Abcam, United Kingdom) and myelin basic protein (MBP; SMI‐94, mouse monoclonal, 1:100; Covance, Princeton, NJ) following a modified protocol.^43^, ^44^, ^45^

Focal areas of complete myelin loss were identified on MBP immuno‐stained sections as demyelinated lesions in the white matter (WML) and gray matter (GML). Cellularity and axonal counts were determined by counting the number of cells in four square ROIs (size: 120 × 120 μm^2^) cast randomly onto WML and nonlesional white matter (NLWM) on hematoxylin and eosin and SMI‐31 stained sections, respectively (Supporting Information Figure S2). Cellularity and axonal counts were expressed as cells per cubic millimeter and axons per cubic millimeter, respectively. Unpaired two‐tailed t‐tests were performed to compare MWF, intracellular/extracellular water fraction (IEWF), and free water fraction (FWF) values in the NLWM and WML ROIs.

## 3 RESULTS

Formalin‐fixed spinal cords of 3 people with MS (1 male, 2 females), and 1 male control subject with no neurological disease, were used. The MS cords were from donors who died at the age of 67–87 years. Their disease duration had been 8–44 years. The MS cords had been fixed for 1127–1441 days. The control subject died at the age of 89 years. Duration of fixation of this cord was 1366 days.

**FIGURE 1:**
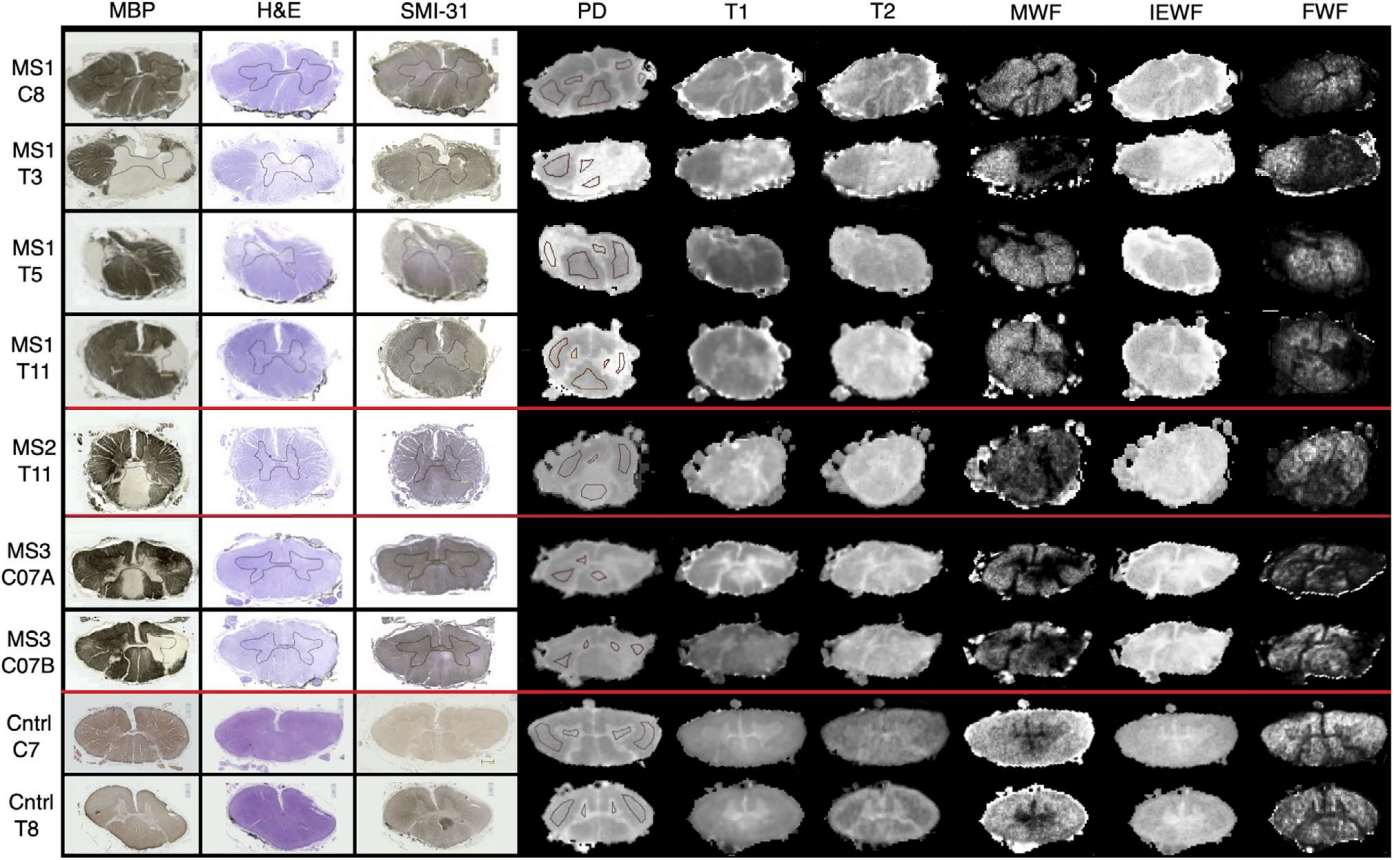
Histology and MR maps of three multiple sclerosis (MS) spinal cords, from top to bottom; sections taken from spinal cord 1 at levels C8, T3, T5 and T11, spinal cord 2 at T11, and spinal cord 3 at two positions in C7. Control cord sections at C8 and T9 are shown on the bottom two rows. The histological slides stained with myelin basic protein (MBP), hematoxylin and eosin (H&E), and SMI at the level of each lesion found are shown next to apparent proton density (aPD), T_1_, T_2_, myelin water fraction (MWF), intracellular/extracellular water fraction (IEWF) and free water fraction (FWF) maps at the same level. From left to right: MBP, H&E, and SMI stained sections, aPD, T_1_, T_2_, MWF, IEWF, and FWF. All images were scaled between identical limits for each contrast so a comparison could be made (aPD 0–5.0 × 10^5^ PU; T_1_ 0–800 ms; T_2_ 10–20 ms; MWF 0%–0.5%; IEWF 0%–1%; and FWF 0%–0.05%). Cntrl, control; PD, proton density

Figure 1 shows matched histology and quantitative MR parameter maps of spinal cords from the 3 MS patients and 1 control subject. Excellent visual correspondence was observed between the anatomic detail on histology images and aPD maps. Both T_1_ and T_2_ maps showed less contrast between lesion and healthy tissue than aPD, whereas the MWF produced high contrast, matching well with the myelin histology in the cords from people with MS. The IEWF map showed, as expected, the inverse of the MWF map (lower in myelinated areas and higher elsewhere). Surprisingly, the FWF map was nonzero in healthy tissue area, especially in the control cords, but decreased to zero in lesion areas. Cord MS454 had no gray‐matter areas that could definitely be determined as normal‐appearing gray matter. Myelin basic protein staining, aPD, MWF, IEWF, and FWF images only are shown together in Supporting Information Figure S3 to facilitate comparison of the maps.

Quantitative histology results and mean values of the MR parameters from the ROIs are given in Table 3. Cellularity and axon count and MBP staining fraction were reduced in lesions compared with normal WM; MWF also showed a decrease in lesion areas, whereas the IEWF increased. We observed a noticeable decrease in the FWF fraction. It should be noted that the MBP staining fraction can only be compared within a single batch; hence, there is variable staining between the pathological cords and the control cord, which was stained at a later date. Therefore, the MBP values were not entered into the correlation analysis for the control cord though it is still included in the table for completeness.

**TABLE 3:** Histology and MR indices for each region of interest

Sample	MBP stain %		Nuclei/mm^2^		Axons/mm^2^		aPD (arb units)		T_1_ (ms)		T_2_ (ms)		MWF %		IEWF %		FWF %	
	WML	NLWM	WML	NLWM	WML	NLWM	WML	NLWM	WML	NLWM	WML	NLWM	WML	NLWM	WML	NLWM	WML	NLWM
MS1 C8	1.78	83.92	1736	1858	7969	20 174	6.0^+05^	4.5^+05^	708.3	445.3	21.4	16	3.6	18.5	96.2	80.4	0.2	1.2
MS1 T3	5.17	78.56	1458	2546	8767	14 601	4.9^+05^	3.4^+05^	518.9	390.6	17.4	14.7	14.1	28.1	85.3	69.7	0.6	2.3
MS1 T5	3.35	87.86	1892	1944	2326	11 782	6.1^+05^	4.1^+05^	604.3	407.2	19.8	15.4	8.7	23.3	91	74.4	0.3	2.3
MS1 T11	1.9	88.31	2760	2778	9514	15 122	5.8^+05^	4.2^+05^	590.9	394.9	19.5	15.2	9	24.9	90.8	76.7	0.2	4.8
MS2 T11	2.89	63.4	2170	2135	7934	12 604	5.6^+05^	4.2^+05^	578.2	517.4	17.4	17.5	13	14.8	86	83	1	2.2
MS3 C07A	15.71	61.46	1215	1615	8281	18 843	5.4^+05^	4.2^+05^	663.6	449.7	18.4	15.4	5.5	23	93.8	75.7	0.7	1.3
MS3 C07B	0.59	43.39	1233	1389	7639	15 625	5.1^+05^	5.0^+05^	634.7	478.9	19.1	15.5	7	18.2	92.6	79.8	0.4	2
Control C7	NA	73.91	NA	27 018	NA	15 634	NA	4.5^+05^	NA	403.5	NA	13.9	NA	29.8	NA	67.1	NA	3.1
Control T8	NA	44.47	NA	27 456	NA	16 571	NA	4.4^+05^	NA	410.4	NA	14.3	NA	29.1	NA	68.5	NA	2.5

Note

There are no lesions in the control cords. Hence, these entries are marked not applicable (NA). All three histology measures were reduced in lesions compared with normal white matter.

**FIGURE 2:**
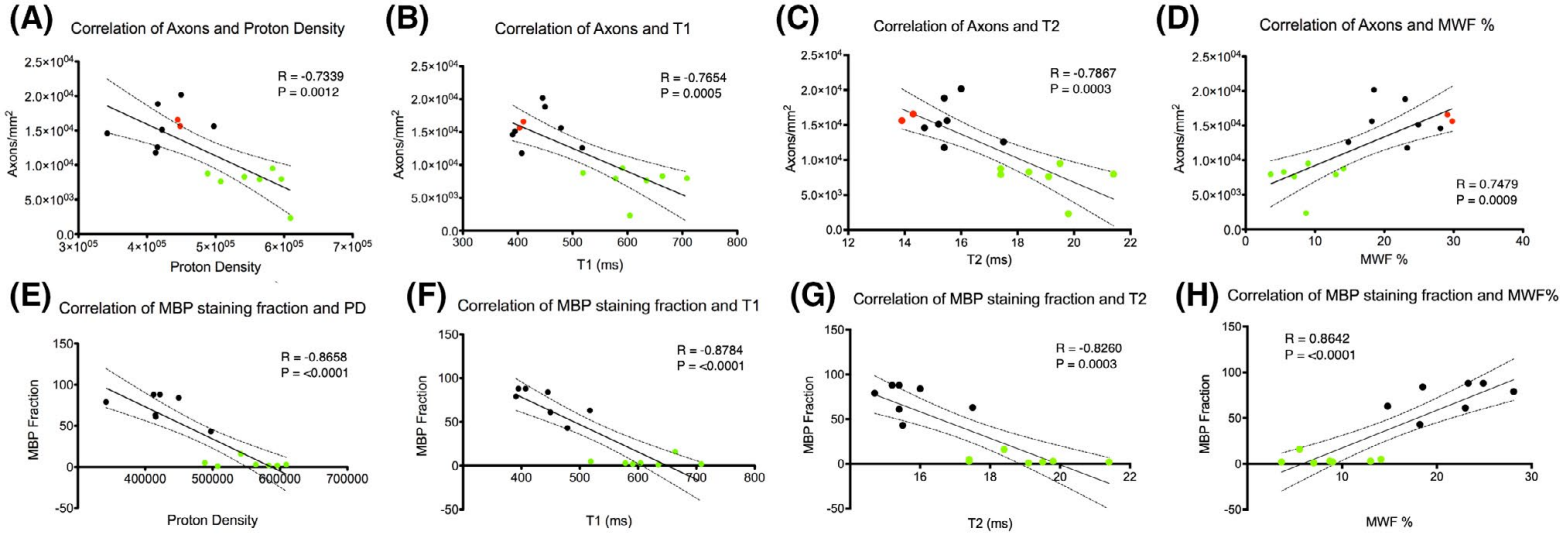
Correlation between histological and MR indices over all white‐matter lesion (WML) and nonlesional white matter (NLWM) in MS cords and control white matter in control cords. Correlation of axons against aPD, T_1_, T_2_, and MWF (A–D, respectively) and MBP against aPD, T_1_, T_2_, and MWF (E–H, respectively). Control cord white‐matter data are shown by the red data points, NLWM by black dots, and WML by green dots. The best fit line is shown with 95% confidence intervals. R, Person’s R value

Graphs of the MWF, IEWF, and FWF values in the NLWM, NLGM, and WM and GM lesion are shown in Supporting Information Figure S4. MWF, IEWF, and FWF were all significantly different between NLWM and WML (*p* < 0.0001). Graphs of correlation (Pearson’s correlation) between histological and MR indices over all WML and NLWM in MS cords, and control WM in control cords (axon counts vs aPD, T_1_, MWF, and MBP stain vs T_1_, MWF) are shown in Figure 2. All are significant (Pearson’s R and *p*‐values found in Supporting Information Table S1). Correlations between MWF and aPD in individual WM types (WML, NLWM, and control WM) and histological indices are shown in Supporting Information Figures S5 and S6, respectively. Correlation between both cell nuclei and axon counts and MWF in WML were not significant. Correlation between nuclei counts and MWF in NLWM and control WM was just significant (*p* = .046), but not significant between axons and MWF in NLWM and control WM. This significance is likely due to the difference in nuclei between control data and NLWM rather than a correlation with MWF.

## 4 DISCUSSION

Our results show that with high‐resolution scanning at 7 T, good correspondence between quantitative MRI and histological metrics is observed. Apparent proton density maps show excellent contrast between healthy and pathological tissue, and T_1_ and T_2_ maps provide quantitative measures that correlate with quantitative histology measures.

The MWF maps show clear differentiation between lesion and nonlesional tissue. The nonzero FWF in the healthy control cord is an unexpected finding, as fixed tissue has not been believed to contain water protons with a long T_2_. This may indicate infiltration by the fixative or may simply be due to bias in the fitting procedure.^31^ The FWF component in the control cords displays better contrast between WM and GM than the MWF, for instance, which could be caused by either of these mechanisms. The measured FWF is lower in lesions in pathological cords, which may also be due to said bias or could indicate the assumption that the FWF is nonexchanging (i.e., that water cannot move between the FWF and intracellular/extracellular water pools) is invalid. In lesions it is possible that biological barriers between these pools break down such that they behave as one, with a common T_1_ and T_2_.

Our samples were not rinsed of fixative before acquisition, as this could cause degradation of the tissue, and therefore contained formaldehyde. Formaldehyde causes known shortening of the T_1_ and T_2_ values, reducing the differences between the myelin and intracellular/extracellular compartments.^46^ The stochastic region contraction search algorithm used in mcDESPOT may not be able to consistently differentiate the signal from the different water pools in this situation. Care was taken to choose appropriate fitting ranges (Table 2), but derived MWF values are highly dependent on the selected parameter ranges, especially when the stochastic region contraction algorithm is used for fitting.^31^, ^47^ An alternate approach such as the Bayesian priors of Bouhrara et al may improve fitting accuracy.^48^ Unfixed tissue is challenging to scan in a suitable time frame after death in humans and may show decomposition over a long scan time; however, if possible, further validation in unfixed tissue would be desirable, as the water pools should exhibit similar separation as in vivo tissue.

The IEWF maps also show good definition between tissue types, with high values in lesional tissue. This implies more space in larger cells and between the cells in these areas. This has been postulated previously by Christiansen et al,^49^ who found that the apparent diffusion coefficient increased in MS lesions and suggested that this may be related to an increase in the extracellular space due to oedema and demyelination. They hypothesized the myelin removed is replaced by water, resulting in an increase of the extracellular space; thereby, the formerly restricted diffusion becomes less restricted.

Despite the issues related to fitting the mcDESPOT model, the MWF and IEWF showed good contrast and anatomic correspondence to the histology images, whereas the single‐component T_1_ and T_2_ maps showed comparatively low contrast. However, the aPD maps also showed excellent correspondence with histology, with increased values in lesion areas. It should be noted that because MWF and IEWF are defined as fractions, the aPD is effectively removed from the mcDESPOT measures; hence, the contrast in the aPD maps is orthogonal information to that in the mcDESPOT parameter maps. Our results imply that if, for example, only the MWF is considered, then an important additional source of information (i.e., the total water content in a voxel) will be missed.

## 5 CONCLUSIONS

High‐resolution quantitative MR parameter maps offer an alternative modality to histology for ex vivo quantitative assessment of MS spinal cord lesions. The MWF maps showed good contrast between lesional and non‐lesional tissue. Proton density, which reflects total water content, correlated significantly in MS lesions and offers additional information compared with the mcDESPOT parameter maps. The mcDESPOT model may benefit from the use of a Bayesian priors algorithm for more challenging conditions.

## CONFLICT OF INTEREST

GJB received honoraria for teaching from GE Healthcare at the time of this study. There are no other known conflicts of interest to disclose.

## Supporting information


**FIGURE S1** Whole spinal cord secured in a glass tube and immersed in perfluoropolyether ready for scanning
**FIGURE S2** Example hematoxylin and eosin (H&E) stained section (left, A) with four square regions of interest (ROIs) positioned in the red outlined region (top right, B); gray matter is outlined in black. Zoomed section (bottom right, C) shows detail in square ROI. Cellularity and axonal counts were determined by counting the number of cells in four square ROIs (size: 120 × 120 μm^2^) cast onto lesional and non‐lesional white matter on the H&E and SMI‐31 stained sections
**FIGURE S3** Expanded from Figure 2, showing only MBP, proton density (PD), myelin water fraction (MWF), intracellular/extracellular water fraction (IEWF), and free water fraction (FWF) to facilitate comparison
**FIGURE S4** Box and whisker plots of all MR parameters in each tissue type. Red points are control samples, for which there are no lesional data. Differences in all parameters were present between nonlesional white matter (NLWM) and white‐matter lesion (WML). Center line is the mean with whiskers of 1 SD
**FIGURE S5** Graphs of correlation for MWF separated into individual white‐matter types expanded from Figure 2: WML and MLWM. Correlations with nuclei against MWF in WML and NLWM in multiple sclerosis (MS) cords and control white matter in control cords (A and C, respectively) and axons against MWF in WLM and NLWM in MS cords and control white matter in control cords (B and D, respectively). Control cord white‐matter data are shown by the red data points. The best fit line is shown with the 95% confidence intervals
**FIGURE S6** Graphs of correlation for proton density separated into individual white‐matter types expanded from Figure 2: WML NLWM. Correlations with nuclei against proton density in WML and NLWM in MS cords and control white matter in control cords (A and C, respectively) and axons against proton density in WML and NLWM in MS cords and control white matter in control cords (B and D, respectively). Control cord white‐matter data are shown by the red data points. The best fit line is shown with the 95% confidence intervals
**TABLE S1** Correlation coefficients for graphs shown in Figure 2

## References

[1] Raine CS . Morphology of myelin and myelination. In: Morell P , ed. Myelin. Springer US; 1984:1‐50.

[2] Morell P , Quarles RH . The myelin sheath. In: Siegel GJ , Agranoff BW , Albers RW , eds. Basic Neurochemistry: Molecular, Cellular and Medical Aspects. Lippincott‐Raven; 1999.

[3] Neumann B , Foerster S , Zhao C , et al. Problems and pitfalls of identifying remyelination in multiple sclerosis. Cell Stem Cell. 2020;26:617‐619. 32386552 10.1016/j.stem.2020.03.017

[4] Katz SI . Classification, diagnosis, and differential diagnosis of multiple sclerosis. Curr Opin Neurol. 2015;28:193‐205. 25887774 10.1097/WCO.0000000000000206

[5] Mowry EM , Pesic M , Grimes B , Deen S , Bacchetti P , Waubant E . Demyelinating events in early multiple sclerosis have inherent severity and recovery. Neurology. 2009;72:602‐608. 19221292 10.1212/01.wnl.0000342458.39625.91 PMC2677540

[6] Arrambide G , Rovira A , Sastre‐Garriga J , et al. Spinal cord lesions: a modest contributor to diagnosis in clinically isolated syndromes but a relevant prognostic factor. Multi Scler. 2018;24:301‐312. 10.1177/1352458517697830 28301287

[7] Okuda DT , Mowry EM , Cree BAC , et al. Asymptomatic spinal cord lesions predict disease progression in radiologically isolated syndrome. Neurology. 2011;76:686. 21270417 10.1212/WNL.0b013e31820d8b1d PMC3053327

[8] Brownlee WJ , Altmann DR , Alves Da Mota P , et al. Association of asymptomatic spinal cord lesions and atrophy with disability 5 years after a clinically isolated syndrome. Multi Scler. 2017;23:665‐674. 10.1177/1352458516663034 27481210

[9] Giovannoni G , Cutter G , Sormani MP , et al. Is multiple sclerosis a length‐dependent central axonopathy? The case for therapeutic lag and the asynchronous progressive MS hypotheses. Mult Scler Relat Disord. 2017;12:70‐78. 28283111 10.1016/j.msard.2017.01.007

[10] Kearney H , Miller DH , Ciccarelli O . Spinal cord MRI in multiple sclerosis—diagnostic, prognostic and clinical value. Nat Rev Neurol. 2015;11:327‐338. 26009002 10.1038/nrneurol.2015.80

[11] Kremenchutzky M , Rice GP , Baskerville J , Wingerchuk DM , Ebers GC . The natural history of multiple sclerosis: a geographically based study 9: observations on the progressive phase of the disease. Brain. 2006;129:584‐594. 16401620 10.1093/brain/awh721

[12] McDonald I , Compston A , et al. Chapter 6: The symptoms and signs of multiple sclerosis. In: Compston A , Confavreux C , Lassmann H , eds. McAlpine’s Multiple Sclerosis, 4th ed. Churchill Livingstone; 2006:287‐346.

[13] Losseff NA , Webb SL , O'Riordan JI , et al. Spinal cord atrophy and disability in multiple sclerosis. a new reproducible and sensitive MRI method with potential to monitor disease progression. Brain. 1996;119:701‐708. 8673483 10.1093/brain/119.3.701

[14] Giorgio A , De Stefano N . Effective utilization of MRI in the diagnosis and management of multiple sclerosis. Neurol Clin. 2018;36:27‐34. 29157402 10.1016/j.ncl.2017.08.013

[15] Schmierer K , McDowell A , Petrova N , Carassiti D , Thomas DL , Miquel ME . Quantifying multiple sclerosis pathology in post mortem spinal cord using MRI. NeuroImage. 2018;182:251‐258. 29373838 10.1016/j.neuroimage.2018.01.052

[16] MacKay A , Whittall K , Adler J , Li D , Paty D , Graeb D . In vivo visualization of myelin water in brain by magnetic resonance. Magn Reson Med. 1994;31:673‐677. 8057820 10.1002/mrm.1910310614

[17] Vavasour IM , Laule C , Li D , et al. Longitudinal changes in myelin water fraction in two MS patients with active disease. J Neurol Sci. 2009;276:49‐53. 18822435 10.1016/j.jns.2008.08.022

[18] MacKay AL , Vavasour IM , Rauscher A , et al. MR relaxation in multiple sclerosis. Neuroimaging Clin N Am. 2009;19:1‐26. 19064196 10.1016/j.nic.2008.09.007

[19] Moore GRW , Leung E , MacKay AL , et al. A pathology‐MRI study of the short‐T2 component in formalin‐fixed multiple sclerosis brain. Neurology. 2000;55:1506‐1510. 11094105 10.1212/wnl.55.10.1506

[20] Laule C , Kozlowski P , Leung E , Li DK , Mackay AL , Moore GR . Myelin water imaging of multiple sclerosis at 7 T: correlations with histopathology. NeuroImage. 2008;40:1575‐1580. 18321730 10.1016/j.neuroimage.2007.12.008

[21] Ljungberg E , Vavasour I , Tam R , et al. Rapid myelin water imaging in human cervical spinal cord. Magn Reson Med. 2017;78:1482‐1487. 28940333 10.1002/mrm.26551

[22] Deoni SC , Rutt BK , Arun T , Pierpaoli C , Jones DK . Gleaning multicomponent T1 and T2 information from steady‐state imaging data. Magn Reson Med. 2008;60:1372‐1387. 19025904 10.1002/mrm.21704

[23] Kitzler HH , Su J , Zeineh M , et al. Deficient MWF mapping in multiple sclerosis using 3D whole‐brain multi‐component relaxation MRI. NeuroImage. 2012;59:2670‐2677. 21920444 10.1016/j.neuroimage.2011.08.052 PMC3673309

[24] Kolind S , Sharma R , Knight S , Johansen‐Berg H , Talbot K , Turner MR . Myelin imaging in amyotrophic and primary lateral sclerosis. Amyotroph Lateral Scler Frontotemporal Degener. 2013;14:562‐573. 23678852 10.3109/21678421.2013.794843 PMC3837681

[25] Deoni SC , Dean DC 3rd , O'Muircheartaigh J , Dirks H , Jerskey BA . Investigating white matter development in infancy and early childhood using myelin water faction and relaxation time mapping. NeuroImage. 2012;63:1038‐1053. 22884937 10.1016/j.neuroimage.2012.07.037 PMC3711836

[26] Deoni SCL , Dean DC , Piryatinsky I , et al. Breastfeeding and early white matter development: a cross‐sectional study. NeuroImage. 2013;82:77‐86. 23721722 10.1016/j.neuroimage.2013.05.090 PMC3777218

[27] Spader HS , Ellermeier A , O'Muircheartaigh J , et al. Advances in myelin imaging with potential clinical application to pediatric imaging. Neurosurg Focus. 2013;34:E9. 10.3171/2013.1.FOCUS12426 PMC3777219 23544415

[28] Miele A , Pan J , Walker L , et al. B‐74 the relationship of myelin content and measures of executive functioning in typically developing children. Arch Clin Neuropsychol. 2014;29:564.

[29] Miele A , Pan J , Walker L , et al. B‐75 neural correlates of emerging executive functioning in 2–5 year olds. Arch Clin Neuropsychol. 2014;29:565.

[30] Lankford CL , Does MD . On the inherent precision of mcDESPOT. Magn Reson Med. 2013;69:127‐136. 22411784 10.1002/mrm.24241 PMC3449046

[31] West DJ , Teixeira R , Wood TC , Hajnal JV , Tournier JD , Malik SJ . Inherent and unpredictable bias in multi‐component DESPOT myelin water fraction estimation. NeuroImage. 2019;195:78‐88. 30930311 10.1016/j.neuroimage.2019.03.049 PMC7100802

[32] Cunniffe N , Coles A . Promoting remyelination in multiple sclerosis. J Neurol. 2021;268:30‐44. 31190170 10.1007/s00415-019-09421-x PMC7815564

[33] Yarnykh VL . Actual flip‐angle imaging in the pulsed steady state: a method for rapid three‐dimensional mapping of the transmitted radiofrequency field. Magn Reson Med. 2007;57:192‐200. 17191242 10.1002/mrm.21120

[34] Wood TC , Simmons C , Hurley SA , et al. Whole‐brain ex‐vivo quantitative MRI of the cuprizone mouse model. Peer J. 2016;4:e2632. 27833805 10.7717/peerj.2632 PMC5101606

[35] Chang LC , Koay CG , Basser PJ , Pierpaoli C . Linear least‐squares method for unbiased estimation of T1 from SPGR signals. Magn Reson Med. 2008;60:496‐501. 18666108 10.1002/mrm.21669 PMC4196213

[36] Stikov N , Boudreau M , Levesque IR , Tardif CL , Barral JK , Pike GB . On the accuracy of T mapping: searching for common ground. Magn Reson Med. 2015;73:514‐522. 24578189 10.1002/mrm.25135

[37] Deoni SC . Transverse relaxation time (T2) mapping in the brain with off‐resonance correction using phase‐cycled steady‐state free precession imaging. J Magn Reson Imaging. 2009;30:411‐417. 19629970 10.1002/jmri.21849

[38] Deoni SC , Matthews L , Kolind SH . One component? Two components? Three? The effect of including a nonexchanging “free” water component in multicomponent driven equilibrium single pulse observation of T1 and T2. Magn Reson Med. 2013;70:147‐154. 22915316 10.1002/mrm.24429 PMC3711852

[39] Tovi M , Ericsson A . Measurements of T1 and T2 over time in formalin‐fixed human whole‐brain specimens. Acta Radiol. 1992;33:400‐404. 1389643

[40] Blamire AM , Rowe JG , Styles P , McDonald B . Optimising imaging parameters for post mortem MR imaging of the human brain. Acta Radiol. 1999;40:593‐597. 10598845 10.3109/02841859909175593

[41] Schmierer K , Wheeler‐Kingshott CAM , Tozer DJ , et al. Quantitative magnetic resonance of postmortem multiple sclerosis brain before and after fixation. Magn Reson Med. 2008;59:268‐277. 18228601 10.1002/mrm.21487 PMC2241759

[42] Bouhrara M , Spencer RG . Incorporation of nonzero echo times in the SPGR and bSSFP signal models used in mcDESPOT. Magn Reson Med. 2015;74:1227‐1235. 26407635 10.1002/mrm.25984 PMC4619140

[43] Geurts JJ , Bo L , Pouwels PJ , Castelijns JA , Polman CH , Barkhof F . Cortical lesions in multiple sclerosis: combined postmortem MR imaging and histopathology. AJNR Am J Neuroradiol. 2005;26:572‐577. 15760868 PMC7976495

[44] Tallantyre EC , Bø L , Al‐Rawashdeh O , et al. Clinico‐pathological evidence that axonal loss underlies disability in progressive multiple sclerosis. Multi Scler. 2010;16:406‐411. 10.1177/1352458510364992 20215480

[45] Petrova N , Carassiti D , Altmann DR , Baker D , Schmierer K . Axonal loss in the multiple sclerosis spinal cord revisited. Brain Pathol. 2018;28:334‐348. 28401686 10.1111/bpa.12516 PMC8028682

[46] Seifert AC , Umphlett M , Hefti M , Fowkes M , Xu J . Formalin tissue fixation biases myelin‐sensitive MRI. Magn Reson Med. 2019;82:1504‐1517. 31125149 10.1002/mrm.27821 PMC6626568

[47] Bouhrara M , Reiter DA , Celik H , Fishbein KW , Kijowski R , Spencer RG . Analysis of mcDESPOT‐ and CPMG‐derived parameter estimates for two‐component nonexchanging systems. Magn Reson Med. 2016;75:2406‐2420. 26140371 10.1002/mrm.25801 PMC5958911

[48] Bouhrara M , Spencer RG . Improved determination of the myelin water fraction in human brain using magnetic resonance imaging through Bayesian analysis of mcDESPOT. NeuroImage. 2016;127:456‐471. 26499810 10.1016/j.neuroimage.2015.10.034 PMC4854306

[49] Christiansen P , Gideon P , Thomsen C , Stubgaard M , Henriksen O , Larsson HB . Increased water self‐diffusion in chronic plaques and in apparently normal white matter in patients with multiple sclerosis. Acta Neurol Scand. 1993;87:195‐199. 8475689 10.1111/j.1600-0404.1993.tb04100.x

