# Supplementary material for: High‐resolution quantitative MRI of multiple sclerosis spinal cord lesions
Source: Magn Reson Med. 2022 Jan 11;87(6):2914–21. doi: 10.1002/mrm.29152 (PMC9208576; doi:10.1002/mrm.29152)
Supplement: Supplementary file 1 — FIGURE S1 Whole spinal cord secured in a glass tube and immersed in perfluoropolyether ready for scanning FIGURE S2 Example hematoxylin and eosin (H&E) stained section (left, A) with four square regions of interest (ROIs) positioned in the red outlined region (top right, B); gray matter is outlined in black. Zoomed section (bottom right, C) shows detail in square ROI. Cellularity and axonal counts were determined by counting the number of cells in four square ROIs (size: 120 × 120 μm2) cast onto lesional and non‐lesional white matter on the H&E and SMI‐31 stained sections FIGURE S3 Expanded from Figure 2, showing only MBP, proton density (PD), myelin water fraction (MWF), intracellular/extracellular water fraction (IEWF), and free water fraction (FWF) to facilitate comparison FIGURE S4 Box and whisker plots of all MR parameters in each tissue type. Red points are control samples, for which there are no lesional data. Differences in all parameters were present between nonlesional white matter (NLWM) and white‐matter lesion (WML). Center line is the mean with whiskers of 1 SD FIGURE S5 Graphs of correlation for MWF separated into individual white‐matter types expanded from Figure 2: WML and MLWM. Correlations with nuclei against MWF in WML and NLWM in multiple sclerosis (MS) cords and control white matter in control cords (A and C, respectively) and axons against MWF in WLM and NLWM in MS cords and control white matter in control cords (B and D, respectively). Control cord white‐matter data are shown by the red data points. The best fit line is shown with the 95% confidence intervals FIGURE S6 Graphs of correlation for proton density separated into individual white‐matter types expanded from Figure 2: WML NLWM. Correlations with nuclei against proton density in WML and NLWM in MS cords and control white matter in control cords (A and C, respectively) and axons against proton density in WML and NLWM in MS cords and control white matter in control cords (B and D, respec [file MRM-87-2914-s001.docx]

**Supporting Information Figure S1**
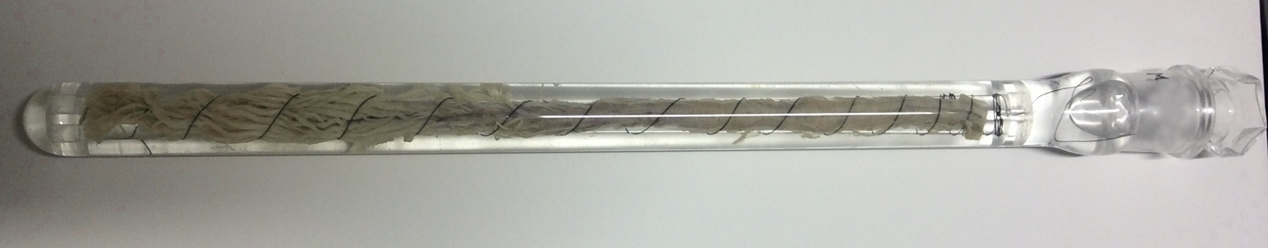


**Supporting Information Figure S1 Caption**

Whole spinal cord secured in a glass tube and immersed in perfluoropolyether ready for scanning.

**Supporting Information Figure S2**
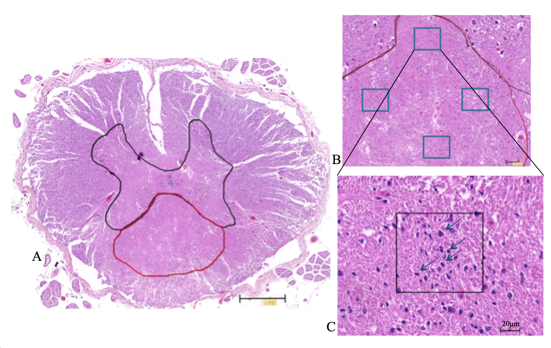


**Supporting Information Figure S2 Caption**

Example H&E stained section (left, A) with four square ROIs positioned in the red outlined region (top right, B), grey matter is outlined in black. Zoomed section (bottom right, C) shows detail in square ROI. Cellularity and axonal counts were determined by counting the number of cells in four square ROIs (size: 120×120μm^2^) cast onto lesional and non-lesional white matter on the H&E and SMI-31 stained sections.

**Supporting Information Figure S3**

**
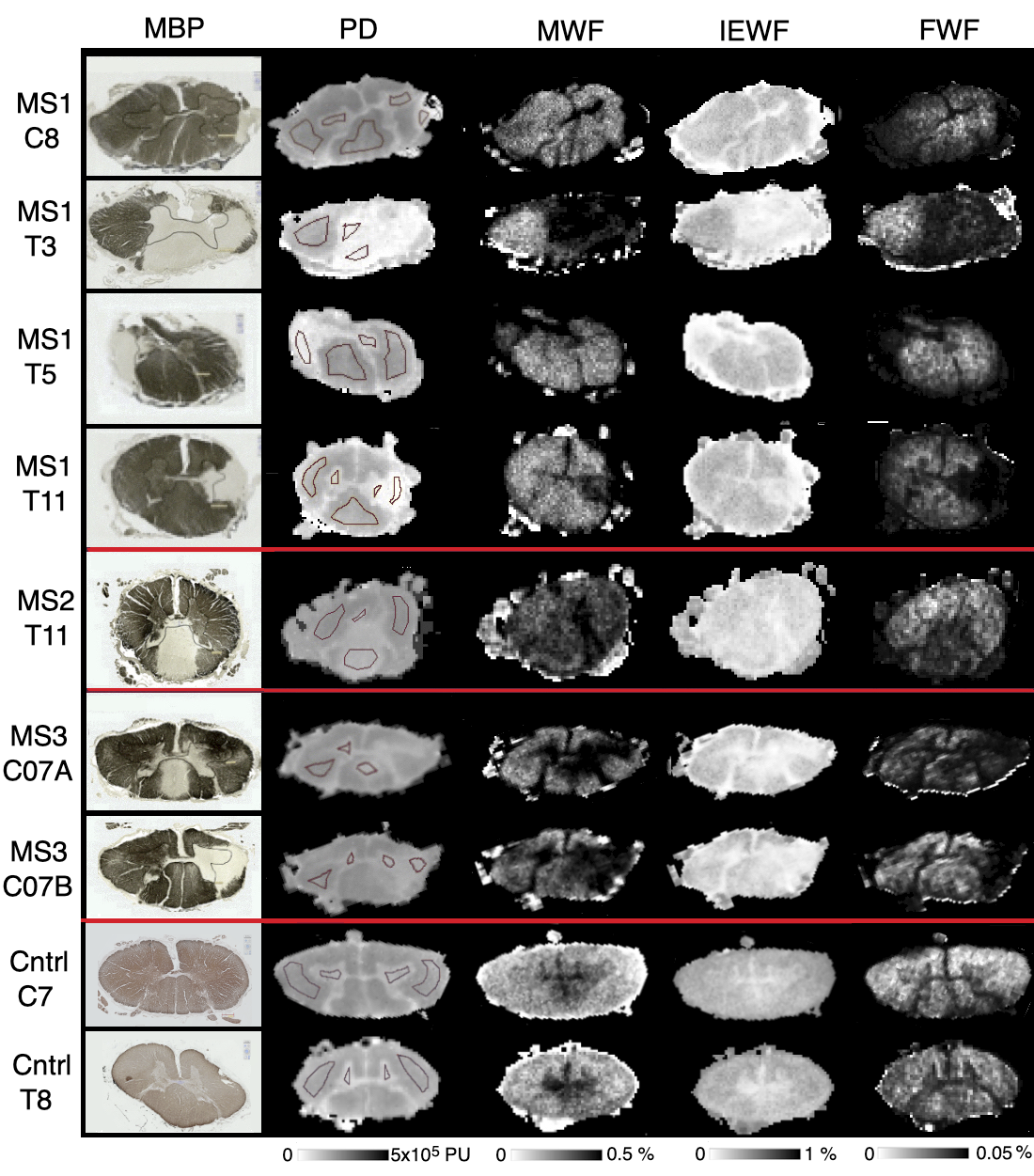
**

**Supporting Information Figure S3 Caption**

Expanded Figure taken from Figure 2 showing only MDP, PD, MWF, IEWF and FWF to facilitate comparison

**Supporting Information Figure S4
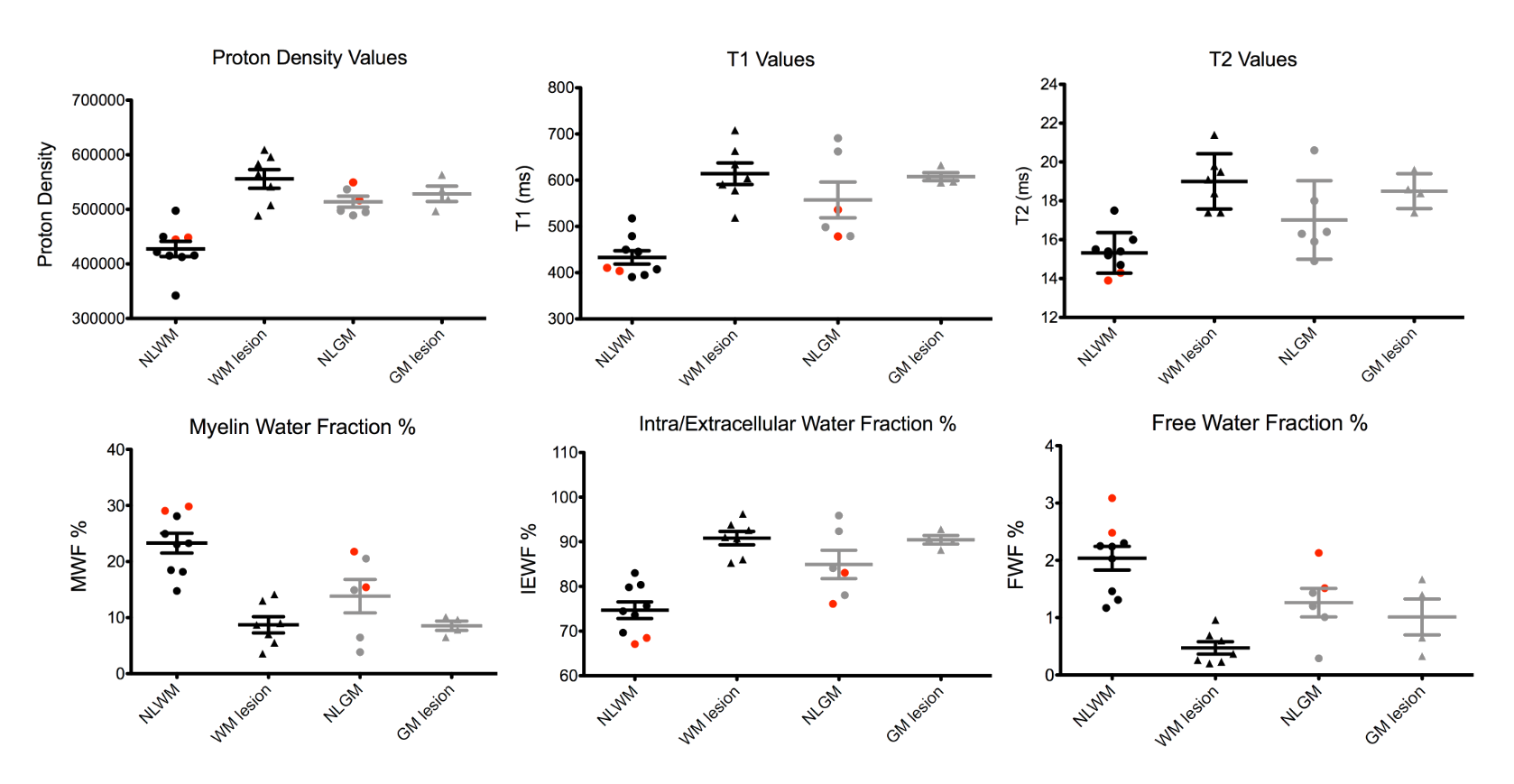
**

**Supporting Information Figure S4 Caption**

Box and whisker plots of all MR parameters in each tissue type. Red points are control samples, for which there is no lesional data. Differences in all parameters were present between NLWM and WM lesion. Centre line is the mean with whiskers of one standard deviation.

**Supporting Information Table S1**

| Histology | MR Indices | R | P |
| --- | --- | --- | --- |
| Axons | Proton Density | -0.7339 | 0.0012 |
|  | T1 (ms) | -0.7654 | 0.0005 |
|  | T2 (ms) | -0.7867 | 0.0003 |
|  | MWF (%) | 0.7479 | 0.0009 |
| MBP Fraction | Proton Density | -0.8658 | <0.0001 |
|  | T1 (ms) | -0.8784 | <0.0001 |
|  | T2 (ms) | -0.8260 | 0.0003 |
|  | MWF (%) | 0.8642 | <0.0001 |

**Supporting Information Table S1 Caption**

Correlation coefficients for graphs shown in Figure 2

**Supporting Information Figure S5**
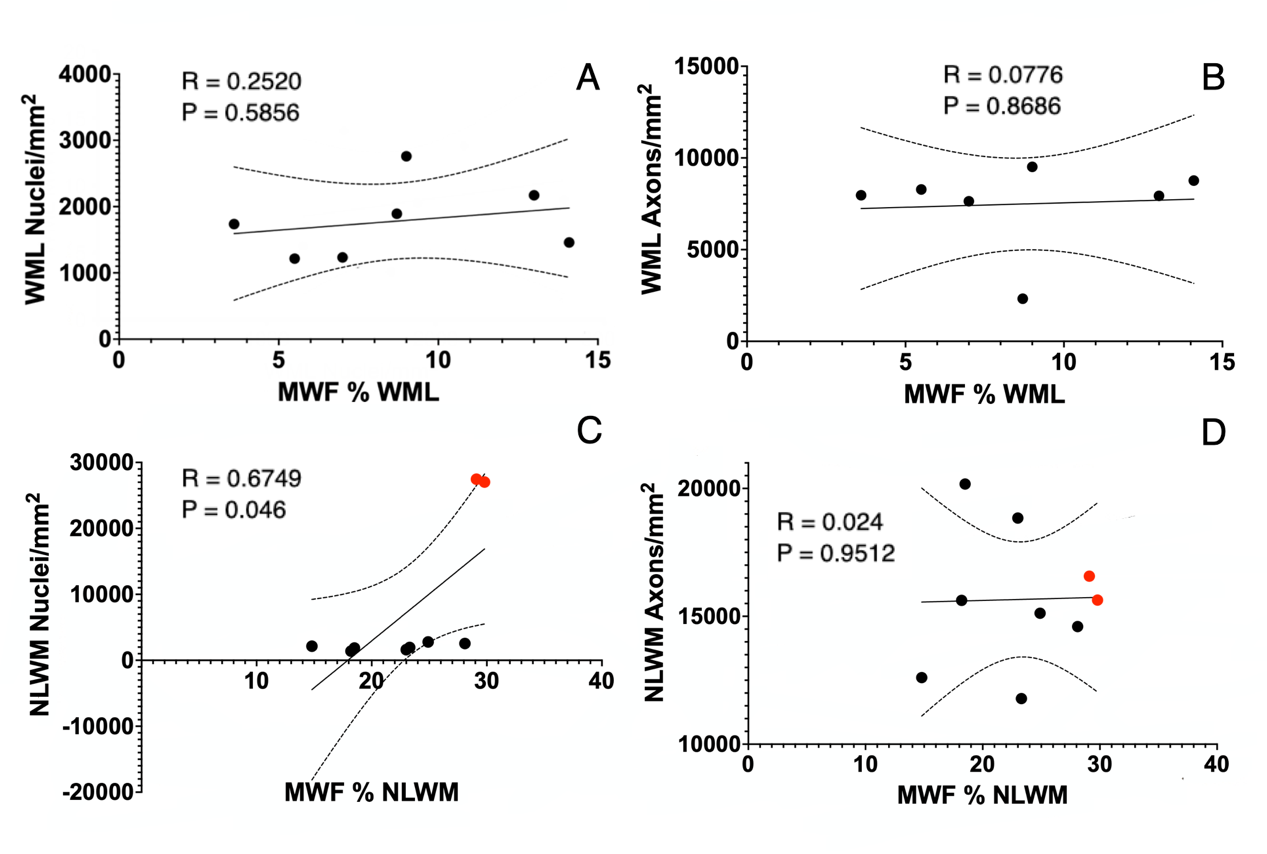


**Supporting Information Figure S5 Caption**

Graphs of correlation for myelin water fraction separated into individual white matter types expanded from Figure 2; white matter lesion and non-lesional white matter. Correlations with nuclei against myelin water fraction in white matter lesion and non-lesional white matter in MS cords and control white matter in control cords (A and C respectively) and axons against myelin water fraction in white matter lesion and non-lesional white matter in MS cords and control white matter in control cords (B and D respectively). Control cord white matter data is shown by the red data points. The best fit line is shown with the 95% confidence intervals.

**Supporting Information Figure S6**


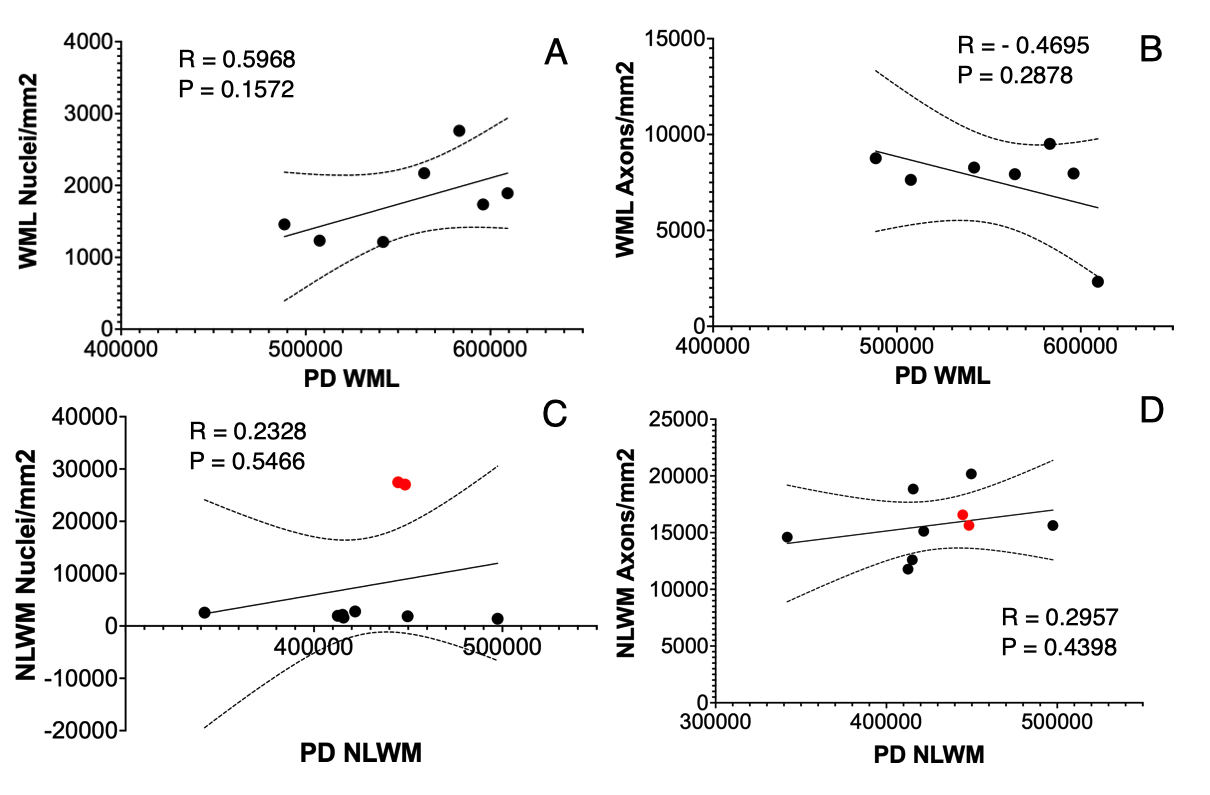


**Supporting Information Figure S6 Caption**

Graphs of correlation for proton density separated into individual white matter types expanded from Figure 2; white matter lesion and non-lesional white matter. There was no significant correlation for PD with histological indices in individual tissue types. Correlations with nuclei against proton density in white matter lesion and non-lesional white matter in MS cords and control white matter in control cords (A and C respectively) and axons against proton density in white matter lesion and non-lesional white matter in MS cords and control white matter in control cords (B and D respectively). Control cord white matter data is shown by the red data points. The best fit line is shown with the 95% confidence intervals.
